# A rare Zinner syndrome combined with testicular cancer: a case report

**DOI:** 10.3389/fonc.2025.1703883

**Published:** 2025-12-02

**Authors:** Linghao Meng, Yanxiang Shao, Yige Jia, Xiangfei Deng, Yaxiong Tang, Xu Hu, Kan Wu, Xiang Li

**Affiliations:** 1 Department of Urology, West China Hospital, Sichuan University, Chengdu, China; 2 Department of Urology, Wusheng County People’s Hospital (Wusheng Hospital Affiliated Hospital of North Sichuan Medical College), Guangan, China

**Keywords:** Zinner syndrome, testicular cancer, surgery, chemotherapy, rare disease

## Abstract

**Background:**

Zinner syndrome is a rare congenital disorder of the urogenital system. In current reports, cases of Zinner syndrome complicated by malignant tumors are even rarer.

**Case description:**

We reported a 26-year-old male with Zinner syndrome complicated by testicular embryonal carcinoma and retroperitoneal lymph node metastasis. He received orchiectomy, chemotherapy and a retroperitoneal lymph node dissection with favorable response.

**Conclusions:**

As suggested by this rare case, Zinner syndrome may be bound up with genitourinary malignancy tumor. When diagnosing congenital disorders of the urinary system, it is essential for clinicians to take into consideration potential complications. More importantly, it is also paramount to account for the similarities and differences in diagnosis and treatment between comorbid and isolated conditions.

## Introduction

Zinner syndrome is a rare male urogenital malformation resulting from abnormal early embryonic development. Zinner first reported this disease in 1914 (1). This syndrome is typically characterized by seminal vesicle cysts, ejaculatory duct obstruction, and absence of the ipsilateral kidney. For this reason, patients may experience multiple symptoms such as difficulty urinating, frequent urination, painful ejaculation, epididymitis, and infertility. This disease is usually diagnosed through imaging tests. Patients with conspicuous symptoms may require surgical intervention (2). This disease is comparatively rare, with approximately 200 cases reported worldwide (3). As a consequence, there is still no unified standard consensus on the diagnosis and treatment of Zinner syndrome. In this case report, we describe a sporadic case of Zinner syndrome combined with a testicular malignant tumor. This case report was written in accordance with the principles outlined in the CARE case report guidelines (https://www.care-statement.org/).

## Case presentation

A 26-year-old male was found to have enlarged retroperitoneal nodules and an absence of the right kidney by employment health examination. The patient had no conscious symptoms throughout the observation period. The patient was previously in desirable health. He is not married and does not have any children. The patient has no history of operations, occupational exposure, infectious diseases, or genetic diseases.

On palpation of the prostate, a mass with unfavorable mobility was detected in the right prostate area. A slight enlargement of the left testicle was observed, with no striking abnormalities in other external genitalia. There was no percussion pain in the bilateral renal areas, and no tenderness in the ureteral and bladder areas.

In laboratory examinations, serum alpha-fetoprotein (AFP) was 58.38 ng/ml (rise), serum β human chorionic gonadotropin (β-HCG) was 1.54 mIU/ml (rise), serum follicle-stimulating hormone (FSH) was 36.9 mIU/ml (rise), serum luteinizing hormone (LH) was 6.4 mIU/ml, serum testosterone was 223 ng/dl, serum inhibin B was 87 pg/ml, serum lactate dehydrogenase (LDH) was 253 U/L (rise), serum carcinoembryonic antigen (CEA) was 0.98 ng/ml, serum creatinine was 84.4 μmol/L, and glomerular filtration rate (GFR) was 110.1 ml/min (**Supplementary Table 1**). Blood routine, urine tests, fecal tests, renal function tests, urine culture, and immunological tests did not show any significant abnormalities.

**Figure 1:**
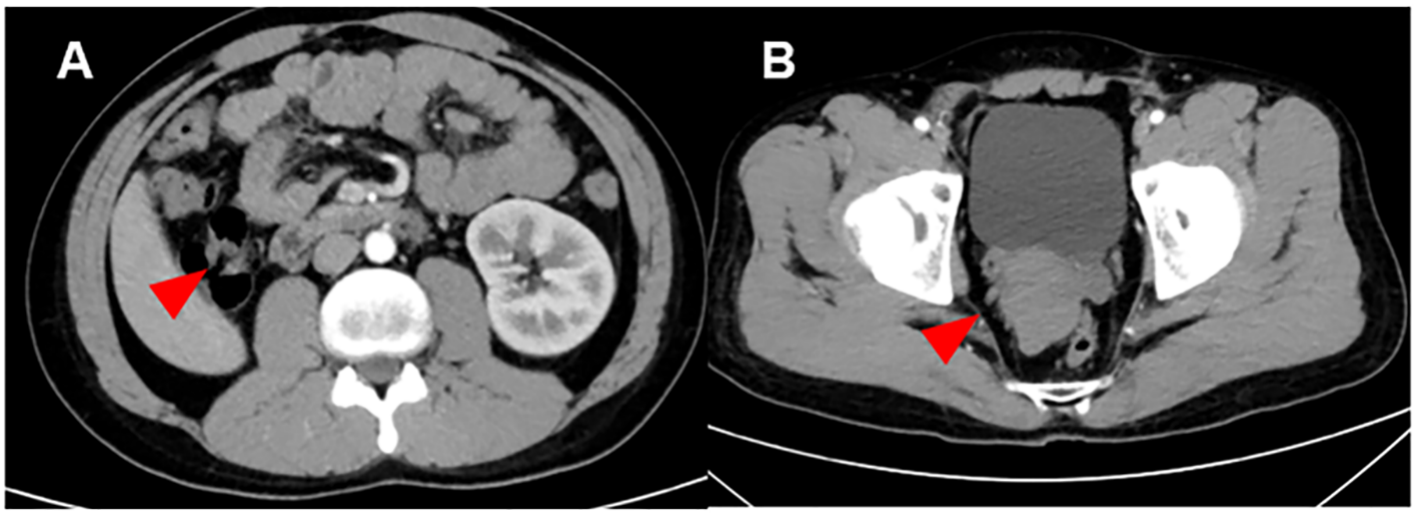
Computed tomography image of the Zinner syndrome. **(A)** Imaging examination showed that the patient has absence of the right kidney: The right kidney and right ureter are not visualized (red arrow). A strip-like soft tissue shadow is seen in the original course area of the right ureter, extending downward to the right seminal vesicle area. **(B)** Imaging examination showed that the patient has seminal vesicle cysts: An irregular mass-like soft tissue shadow is seen in the right seminal vesicle area (red arrow), measuring approximately 6.4 × 4.6 cm, with no definite enhancement. It is closely adjacent to the posterior wall of the bladder, the prostate, and the left seminal vesicle. The right seminal vesicle with its normal morphology is not visualized.

**Figure 2:**
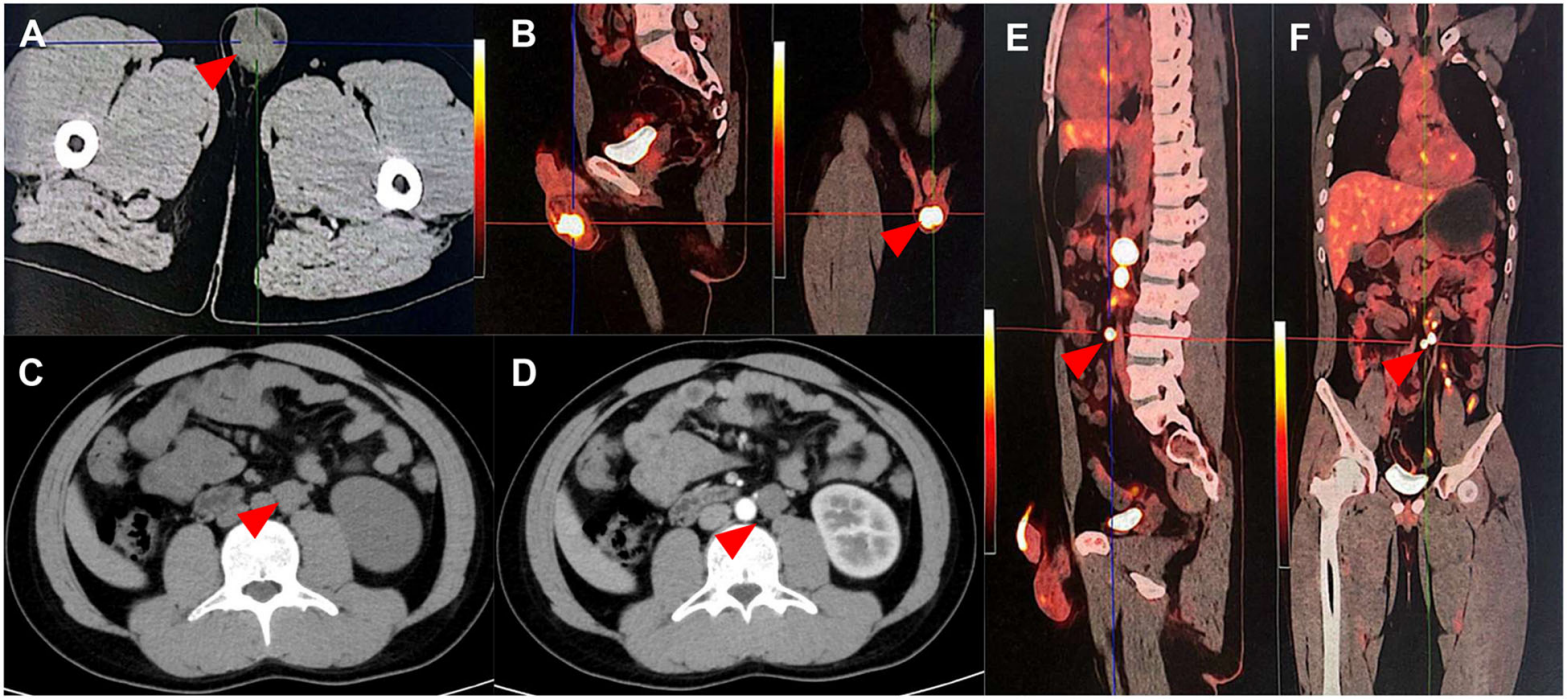
Computed tomography and Positron Emission Tomography and Computed Tomography image of the testicular cancer. **(A, B)** Primary lesion of the testicular cancer: The left testis is enlarged with a soft tissue density nodule, measuring approximately 2.92 cm × 2.81 cm (red arrow). The 18F-FDG radioactive uptake is significantly enhanced, with a maximum standardized uptake value (SUV max) of 13.80. **(C–F)** Retroperitoneal lymph node metastatic lesion of the testicular cancer: Multiple enlarged lymph nodes of varying sizes are detected adjacent to the abdominal aorta and left iliac vessels, with varying degrees of increased radioactive uptake. The SUV max ranges from 7.04 to 31.71, and the larger one measures approximately 28.9 mm × 21.4 mm.

As Enhanced Computed Tomography (CT) suggested, the patient had an absent right kidney and an irregular mass measuring 6.4 × 4.6 cm in the right seminal vesicle area, immediately behind the posterior wall of the bladder, the prostate, the rectum, and the left seminal vesicle, from which no significant enhancement was observed (**Figure 1**). Ultrasound examination revealed a slight enlargement of the left testis, with a volume of approximately 24.6 ml and heterogeneous hypoechoic parenchyma; the right testis had a volume of about 15.2 ml and the echo of the testicular parenchyma is relatively homogeneous. No remarkable testicular microlithiasis was detected in either testis. Positron Emission Tomography and Computed Tomography (PET-CT) suggested a left testicular tumor, measuring approximately 2.92 × 2.81 cm, and multiple enlarged lymph nodes were observed adjacent to the abdominal aorta and the left iliac vessels, which demonstrated a strikingly elevated radioactivity uptake (**Figure 2**). The patient’s semen tests suggested the absence of sperm cells. No any abnormalities were observed throughout Y chromosome microdeletion tests and karyotyping tests.

The patient was diagnosed with Zinner syndrome with testicular cancer and retroperitoneal lymph node metastasis.

**Figure 3:**
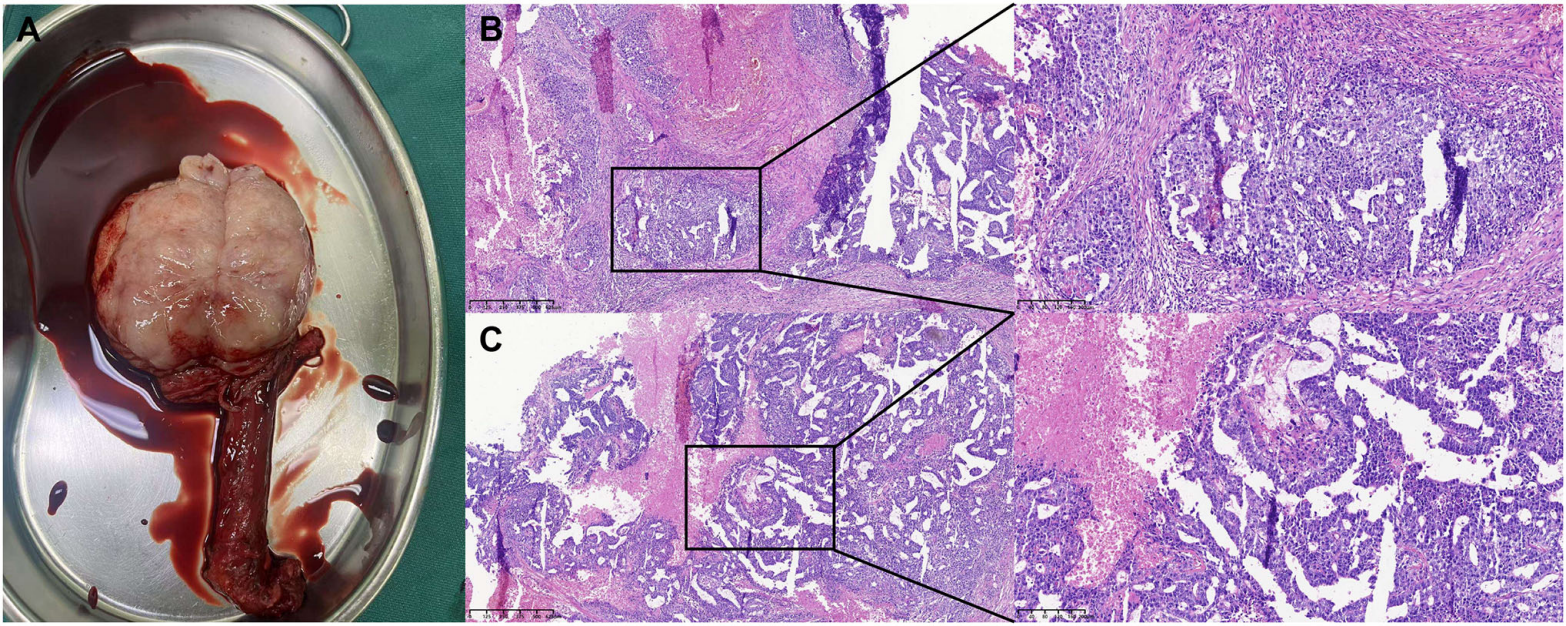
Surgical resection specimen and pathological staining of the testicular cancer. **(A)** Surgical resection specimen of the testicular cancer. **(B, C)** Postoperative pathology examination suggested testicular embryonal carcinoma: No cancer was detected at the spermatic cord stump.

The patient was treated with a radical orchiectomy of the left testis. The immunohistochemistry revealed SALL4 (+), OCT3/4 (+), CD30 (+), PCK (+), EMA (–), PLAP (+), CD117 (+/-), GPC3 (+), AFP (–), Ki67 (80% +), and comply with embryonal carcinoma. No mixed germ cell tumor was identified in the pathological sections. In addition, there was no tumor invasion in the residual end of the spermatic cord. Germ cell neoplasia *in situ* has not been detected. In the excised testicular specimen, we did not detect obvious signs of testicular hypoplasia or seminiferous tubule dysplasia. Aside from that, no conspicuous tubules underwent spermatogenesis. Moreover, some tubules containing only Sertoli cells were detected (**Figure 3**, **Supplementary Table 2**). The patient received three cycles of standard adjuvant BEP (Bleomycin, Etoposide, and Cisplatin) chemotherapy after orchiectomy. AFP was 3.36 ng/ml, and β-HCG could not be detected subsequent to chemotherapy. The volume of the enlarged lymph nodes decreased in the aftermath of chemotherapy. He subsequently received a retroperitoneal lymph node dissection (RPLND). A postoperative examination of the lymph nodes suggested necrotic tissue, and no residual tumor tissue was observed. It is noteworthy that the patient has not experienced symptoms that seriously affect their quality of life. In accordance with the patient’s wishes, we thereby have temporarily refrained from further intervention for the patient’s seminal vesicle cyst and reproductive impairment. If the patient has fertility needs, we will further evaluate and intervene in the patient’s infertility in the subsequent period. The patient is currently undergoing postoperative follow-up.

## Discussion

As a rare congenital disorder of the genitourinary system, Zinner syndrome records a global incidence rate of approximately 0.005% (4). Zinner syndrome is characterized by seminal vesicle cysts, ipsilateral renal hypoplasia, and ejaculatory duct obstruction. The organs of the urogenital system develop from the mesonephric duct. The distal end of the mesonephric duct divides to form the bladder neck, seminal vesicles, vas deferens, ejaculatory ducts, and epididymis (5). The ureteric bud and the posterior kidney germ layer fuse to form the primitive kidney (6). Abnormalities in this developmental process can give rise to malformations of the urogenital system. In early embryonic stages, Zinner syndrome is associated with abnormal development of the mesonephric duct, ultimately bringing about abnormal development of the kidney and ipsilateral germ-derived structures.

Patients with Zinner syndrome typically suffer from a wide range of symptoms such as difficulty urination, frequent urination, perineal pain, epididymitis, and painful ejaculation. Attributable to obstruction of the ejaculatory ducts, patients may endure oligospermia or azoospermia, and 45% of patients will suffer infertility (7). Rooted in 52 cases of Zinner syndrome, a case report analysis illustrated that lesions were more common on the right side (5). When the seminal vesicle cyst is small, the patient may not experience any symptoms. This patient had no conspicuous symptoms. During a health examination, he was found to have a missing right kidney and a seminal vesicle cyst. Worse still, subsequent sperm analysis revealed azoospermia.

Zinner syndrome can usually be detected through digital rectal examination and imaging tests. This patient was screened for right kidney agenesis through ultrasound examination. Ultrasound examination is featured by non-invasiveness and radiation-absence, positioning it as a suitable option for screening for Zinner syndrome (8). Ultrasound is favorably characterized by superior diagnostic value for bleeding or infection in seminal vesicle cysts. CT can provide a more intuitive view of the association between seminal vesicle cysts and surrounding tissue structures (9). Under such circumstances, a full abdominal CT scan further confirmed the diagnosis of Zinner syndrome. Typical CT findings include unilateral renal agenesis combined with multiple cysts in the ipsilateral seminal vesicle. It is paramount to mention that the cysts exert noticeable pressure on one side of the posterior wall of the bladder. The seminal vesicle cysts are round, with slightly irregular borders or obvious tubular dilatation. Calcification and hemorrhage may be present. In general, dilated vas deferens, ejaculatory ducts, and ectopic dilated residual ureters communicating with the seminal vesicles can be observed. Magnetic Resonance Imaging (MRI) is universally acknowledged for its striking resolution for soft tissue and is the best imaging method for assessing anatomical relationships in the pelvic region (10).

For the time being, there is no standard treatment for Zinner syndrome. Most interventions are symptomatic treatments (11). Other than abnormal sperm examination results, this patient did not experience any remarkable discomfort. Ascribable to the fact that the patient also had a malignant tumor, we have not yet conducted excessive interventions for Zinner syndrome. Hence, surgical treatment should be actively applied when patients have conspicuous symptoms or conservative treatment is ineffective. For large seminal vesicle cysts, laparoscopic surgery is currently the most extensively utilized surgical method (12–14). Laparoscopic surgery is characterized by minimal trauma, minimal bleeding, and rapid recovery. Attributable to the deep anatomical location of the seminal vesicles, laparoscopic surgery has multiple advantages over conventional open surgery.

Case reports of Zinner syndrome combined with malignant tumors are extremely rare. Bhat et al. reported a case of Zinner syndrome combined with primary seminal vesicle adenocarcinoma (15). They suggest that ectopic ureter and renal hypoplasia expose the epithelial tissue of the seminal vesicles to carcinogens, ultimately resulting in malignant transformation of seminal vesicle cysts (16). The postoperative pathology of this patient suggested unfavorably differentiated adenocarcinoma. After surgery, the patient received the adjuvant chemotherapy regimen of carboplatin plus paclitaxel. A recurrence occurred postoperatively, and the patient achieved clinical remission after receiving additional chemotherapy. Sato et al. reported a case of Zinner syndrome integrated with a malignant prostate tumor (17). In this case, the pathological type of the tumor is renal-type clear cell carcinoma. The patient received adjuvant treatment with sunitinib after surgery and died of multiple organ failures at 29 months postoperatively. They speculated that the underdeveloped ectopic kidney tissue underwent malignant transformation in old age. Khoda et al. have described the incidental discovery of Zinner syndrome in testicular germ cell tumors (18). Similar to our case, this patient received postoperative adjuvant chemotherapy. They underscored the imperative to concentrate on anatomical abnormalities stemming from Zinner syndrome during retroperitoneal lymph node dissection for testicular cancer. As jointly suggested by these case reports, the occurrence of these malignant tumors may be correlated with congenital developmental abnormalities.

Throughout embryonic development, both the testes and the urinary system develop from the mesoderm. The patient we reported suffers from Zinner syndrome combined with testicular tumors, which may be associated with abnormal development of the mesoderm during the embryonic period or external stimuli. Despite the fact that Zinner syndrome has a low incidence rate, it remains a contributor to the development of malignant tumors in the genitourinary system. Malignancy may arise from the residual embryonic components and persistent inflammatory stimulation (19).

Radical orchiectomy serves as the cornerstone of treatment for malignant testicular tumors, and this procedure can provide crucial histopathological information (20). The pathological type of this testicular tumor is nonseminomatous germ cell tumors (NSGCTs). Adjuvant chemotherapy after surgery holds profound significance and necessity for NSGCTs. Chemotherapy for NSGCTs is usually grounded in platinum-based chemotherapeutic agents. Common chemotherapy regimens include BEP (bleomycin, etoposide, cisplatin) and EP (etoposide, cisplatin). For NSGCTs with lymphovascular invasion, postoperative chemotherapy can lower the 5-year recurrence rate to 3%, thereby strikingly lowering the risk of recurrence (21). This patient received three cycles of BEP regimen chemotherapy after radical orchiectomy. Considering that the patient still had residual enlarged retroperitoneal lymph nodes after receiving chemotherapy, the patient subsequently underwent RPLND. RPLND that preserves the postganglionic sympathetic nerves of the lumbar nerves has been widely used. This technique enables most patients to maintain antegrade ejaculation, and its cure rate is comparable to that of traditional surgery (22). As already evidenced by relevant studies, chemotherapy combined with RPLND can bring about a 10-year recurrence-free survival rate of 98% for patients (23). Fortunately, the retroperitoneal lymph nodes revealed only necrotic tissue present and no malignant components detected after chemotherapy. Under such circumstances, it remains a paramount problem with regard to whether the patient’s clinical remission is bound up with the Zinner syndrome, which entails an in-depth exploration. On top of that, whether congenital genitourinary system developmental abnormalities are associated with the development of malignant tumors necessitates profound investigation and assessment. In the future, it may be essential to conduct precise genetic testing, so as to assess the treatment and prognosis for such cases.

## Data Availability

The original contributions presented in the study are included in the article/**Supplementary Material**. Further inquiries can be directed to the corresponding author/s.
