# Supplementary material for: A rare Zinner syndrome combined with testicular cancer: a case report
Source: Front Oncol. 2025 Dec 2;15:1703883. doi: 10.3389/fonc.2025.1703883 (PMC12705352; doi:10.3389/fonc.2025.1703883)
Supplement: Supplementary file 1 [file Table1.docx]

**Supplementary Materials**

**Supplementary Table 1: Laboratory Test Results and Reference Ranges of the Patient**

| **Project** | **Abbreviation** | **Value** | **Normal Reference Range** | **Unit** | **Status** |
| --- | --- | --- | --- | --- | --- |
| Alpha-fetoprotein | AFP | 58.38 | 0.00 ~ 13.20 | ng/ml | **rise** |
| β human chorionic gonadotropin | β-HCG | 1.54 | 0.22 ~ 0.92 | mIU/ml | **rise** |
| Follicle-stimulating hormone | FSH | 36.9 | 1.5 ~ 12.4 | mIU/ml | **rise** |
| Luteinizing hormone | LH | 6.4 | 2.4 ~ 12.9 | mIU/ml | normal |
| Testosterone | T | 223.00 | 142.39 ~ 923.14 | ng/dl | normal |
| Inhibin B | Inhibin B | 87 | 80 ~ 350 | pg/ml | normal |
| Lactate dehydrogenase | LDH | 253 | 120 ~ 250 | U/L | **rise** |
| Carcinoembryonic antigen | CEA | 0.98 | 0.00 ~ 4.50 | ng/ml | normal |
| Creatinine | Cr | 84.4 | 57.0 ~ 97.0 | μmol/L | normal |
| Glomerular filtration rate | GFR | 110.1 | - | ml/min | normal |

*The test results were based on the patient's serum sample.*

**Supplementary Table 2: Information on Antibodies Used for Immunohistochemical Staining**

| **Target** | **Manufacturer** | **Catalog Number** | **Host Species** | **Antibody Type** |
| --- | --- | --- | --- | --- |
| SALL4 | Roche Diagnostics, USA | 760-4864 | Mouse | Monoclonal |
| OCT3/4 | Leica Biosystems Newcastle Ltd, UK | NCL-L-OCT3/4 | Mouse | Monoclonal |
| CD30 | DAKO (Agilent) A/S, Denmark | M0751 | Mouse | Monoclonal |
| PCK | Roche Diagnostics, USA | 760-2135 | Mouse | Monoclonal |
| EMA | Roche Diagnostics, USA | 790-4463 | Mouse | Monoclonal |
| PLAP | Roche Diagnostics, USA | 760-2664 | Mouse | Monoclonal |
| CD117 | Cell Marque Corporation, USA | 117R-16 | Rabbit | Monoclonal |
| GPC3 | Roche Diagnostics, USA | 790-4564 | Mouse | Monoclonal |
| AFP | Leica Biosystems Newcastle Ltd, USA | NCL-AFP | Mouse | Monoclonal |
| Ki67 | Cell Marque Corporation, USA | 275R-16 | Rabbit | Monoclonal |
